# Real world study of GLP-1 receptor agonists in overweight or obese type 2 diabetes by using repeated measurement analysis of variance

**DOI:** 10.1097/MD.0000000000038879

**Published:** 2024-08-09

**Authors:** Wanying Yang, Xiangming Zhou, Yuanyuan Miao, Lu Wang, Yunhui Zhao, Tingyu Ke, Lili Ban

**Affiliations:** a Department of Pharmacy, The Second Affiliated Hospital of Kunming Medical University, Kunming, Yunnan, China; b Department of Endocrinology, The Second Affiliated Hospital of Kunming Medical University, Kunming, Yunnan, China.

**Keywords:** GLP-1 receptor agonist, obesity, overweight, repeated measurement analysis of variance, type 2 diabetes mellitus

## Abstract

To investigate the safety and efficacy of long-acting glucagon like peptide-1 receptor agonists in overweight or obese patients with type 2 diabetes. Overweight or obese patients with type 2 diabetes from July 2021 to June 2022 were randomly divided into control group (metformin) and experimental group (metformin + dulaglitide or semaglutide). Repeated measures analysis of variance was used to compare Hemoglobin A1c, fasting plasma glucose and body mass index (BMI) of patients before treatment, 6 months and 12 months after treatment. The adverse reactions of patients before treatment and 12 months after treatment were analyzed. The time effect of Hemoglobin A1c, fasting plasma glucose and BMI in the control group (n = 35) and the experimental group (n = 32) were statistically significant (*P* < .001), and the intergroup effect of BMI was statistically significant (*P* < .05). The interaction effect of BMI was statistically significant (*P* < .001). The BMI level of the experimental group was lower than that of the control group at 6 and 12 months after treatment (*P* < .001). There was no significant difference in the incidence of adverse reactions between the 2 groups (*P* > .05). Long-acting glucagon like peptide-1 receptor agonists, such as dulaglitide and semaglutide, not only reduce glycosylated hemoglobin levels, but also significantly improve BMI in overweight or obese patients with type 2 diabetes.

## 1. Introduction

With the accelerated pace of life in modern society, the prevalence of type 2 diabetes mellitus in overweight or obese people is increasing year by year, thus increasing the risk of cardiovascular disease.^[1]^ Glucagon like peptide-1 (GLP-1) receptor agonist can reduce glucose in a glucose concentration dependent manner by simulating the activation of natural GLP-1 receptor, while simultaneously reducing weight, lowering blood pressure, and improving blood lipid profile.^[2]^ As a new hypoglycemic drug, GLP-1 receptor agonist is currently receiving more and more clinical attention. However, the follow-up time of previous studies on its treatment of overweight or obese type 2 diabetes mellitus is usually short, especially the previous studies only set a single observation node,^[3,4]^ which may lead to relatively one-sided results. Repeated measures analysis of variance can effectively use the data of each time node, fully consider the trend of each indicator over time and the possible interaction effect between them, so that the results are relatively more objective.^[5,6]^ Based on repeated measurement analysis of variance, this study followed up for 1 year to observe the safety and effectiveness of long-acting GLP-1 receptor agonist in the treatment of overweight or obese type 2 diabetes mellitus, providing reference value for its further clinical rational use.^[7,8]^

## 2. Objects and methods

### 2.1. The subject of the study

Inclusion criteria: (1) comply with the diagnostic criteria for type 2 diabetes established by the American Diabetes Association in 2020; (2) body mass index (BMI) ≥ 24 kg/m^2^; (3) 20 to 80 years old; (4) patients or their caregivers can communicate in language or words. Exclusion criteria: (1) previous use of GLP-1 receptor agonists; (2) acute complications of diabetes mellitus; (3) combined with digestive system and other serious diseases, infections or malignant tumors; (4) pregnant or lactating women.

According to the inclusion and exclusion criteria, patients with type 2 diabetes who visited the national Metabolic Management Center (MMC) in the Second Affiliated Hospital of Kunming Medical University from July 2021 to June 2022 were selected as the research objects. The patient signed the informed consent form based on MMC platform, and the research scheme was reviewed and approved by the hospital ethics committee.

### 2.2. Treatment

The subjects were randomly divided into 2 groups by random number table. The control group was treated with metformin, and the test group was treated with dulaglutide [Vetter Pharma-Fertigung GmbH & Co. KG, registration number S20190022], 1.5 mg, once a week; or semaglutide [Novo Nordisk, Denmark, National Drug Approval No. SJ20210015], the dose is 0.5 mg, once a week, and the maximum dose is 1 mg per week.

Both groups adopted MMC management mode, mainly including (1) enrollment: establishing files for patients, conducting questionnaire survey, understanding patients’ conditions in disease cognition, blood glucose monitoring, drug treatment and lifestyle, and recording relevant in-patient or outpatient inspection reports and laboratory indicators. (2) Follow-up: follow up by phone every 3 months, and notify the patient to return to MMC by phone every 6 months to review relevant indicators. (3) Health education: a multidisciplinary team composed of diabetes specialists, nurses, pharmacists, and nutritionists in MMC will regularly give face-to-face lectures or individualized health education to patients.

### 2.3. Effectiveness ratings

The main efficacy indicators were Hemoglobin A1c (HbA1c), fasting plasma glucose (FPG) and BMI.^[9,10]^ The primary and secondary efficacy indicators were observed before treatment, 6 months after treatment and 12 months after treatment. In addition, the safety of the drug was evaluated, mainly observing whether the patients had any adverse reactions such as hypoglycemia and gastrointestinal discomfort after 12 months of treatment.

### 2.4. Statistical methods

The data were analyzed by SPSS 22.0 statistical software. The measurement data conforming to the normal distribution are expressed as mean ± standard deviation, and the inter group comparison is performed by *t* test; the counting data were expressed by rate or constituent ratio, and the comparison between groups was performed by $\chi^{2}$ test. In the repeated measurement variance analysis, if the Mauchly’s spherical hypothesis test is not satisfied, read the results of Greenhouse Geisser method; when there is no interaction effect between time and treatment factors, the main effect test is used to evaluate the effect of treatment factors. *P* < .05 indicates that the difference is statistically significant; If there is interaction between time and processing factors, the Bonferroni method is used to test the level α = 0.05 after correction, compare 2 pairs.

## 3. Results

### 3.1. General information

A total of 70 cases were included in this study, 35 in each of the 2 groups. Because 3 patients in the test group withdrew due to economic reasons within 6 months after treatment, 35 patients in the control group and 32 patients in the test group were finally included in the statistical analysis. The average age of the control group was 54.57 ± 10.05 years old, and the average course of disease was 7.87 ± 3.86 years, including 19 males and 16 females; the average age of the test group was 55.81 ± 10.91 years old, and the average course of disease was 6.68 ± 3.60 years, including 18 males and 14 females. There was no statistically significant difference between the 2 groups in the baseline indicators (*P* > .05). See Table 1 for details.

**Table 1:** Compare 2 groups of patients with baseline data.

Variable	Control group (n = 35)	Experimental group (n = 32)	$\chi^{2}/t$ value	*P* value
Sex/case (%)			0.026	.872
Male	19 (54.29)	18 (56.25)		
Female	16 (45.71)	14 (43.75)		
Age/years	54.57 ± 10.05	55.81 ± 10.91	‐0.485	.629
Duration of illness/year	7.87 ± 3.86	6.68 ± 3.60	1.291	.201
BMI/kg·m^-2^	27.74 ± 2.33	27.77 ± 3.25	‐0.042	.967
HbA1c/%	8.98 ± 1.48	8.58 ± 1.68	1.034	.305
FPG/mmol·L^-1^	8.33 ± 2.24	7.98 ± 2.36	0.615	.541
TC/(mmol·L^-1^)	5.64 ± 1.87	5.44 ± 1.56	1.751	.085
TG/(mmol·L^-1^)	2.62 ± 1.11	2.15 ± 0.77	1.984	.052

BMI **=** body mass index, FPG = fasting plasma glucose, HbA1c = Hemoglobin A1c, TC = serum total cholesterol, TG = serum triglyceride.

### 3.2. Comparison of effects of BMI, HbA1c, and FPG

**Figure 1.**
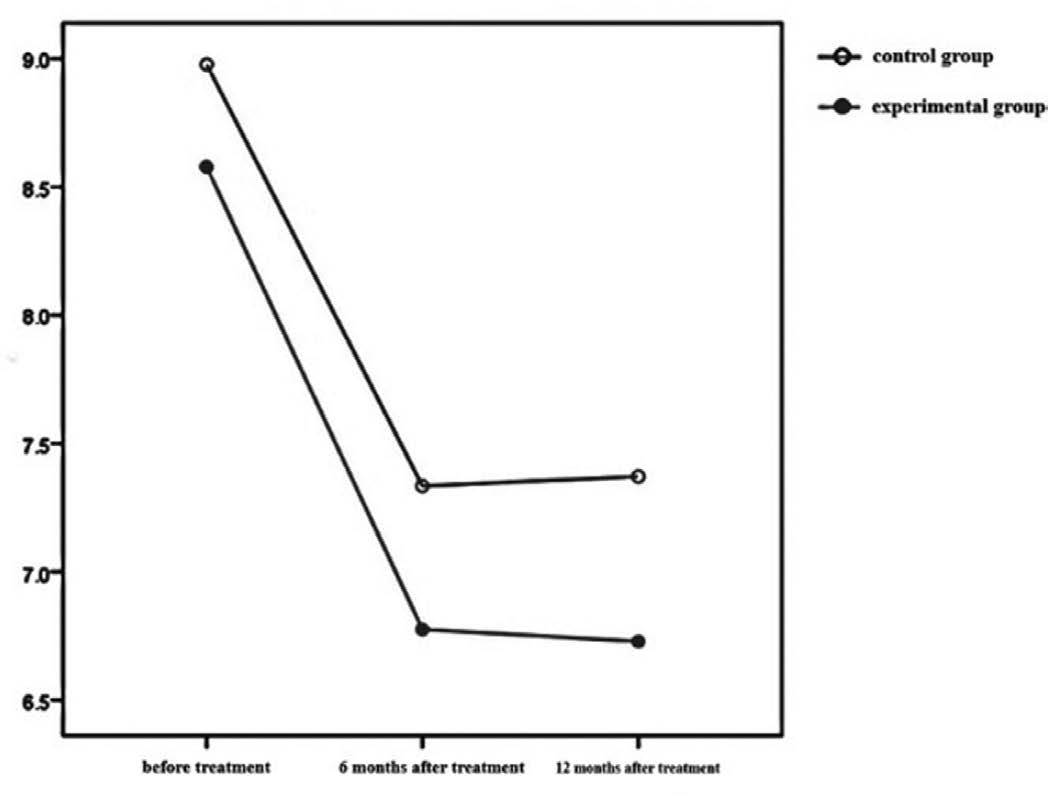
HbA1c levels at different time points and interaction by group in the 2 groups. HbA1c = Hemoglobin A1c.

**Figure 2:**
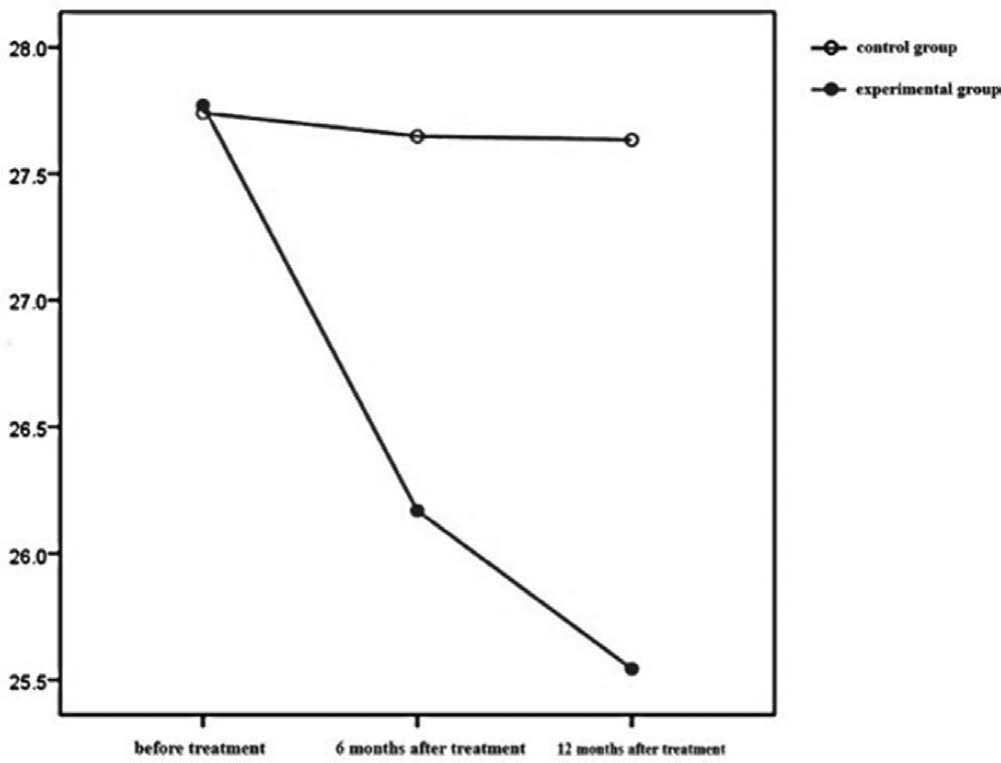
. BMI levels at different time points and interaction by group in the 2 groups. BMI = body mass index.

**Figure 3.**
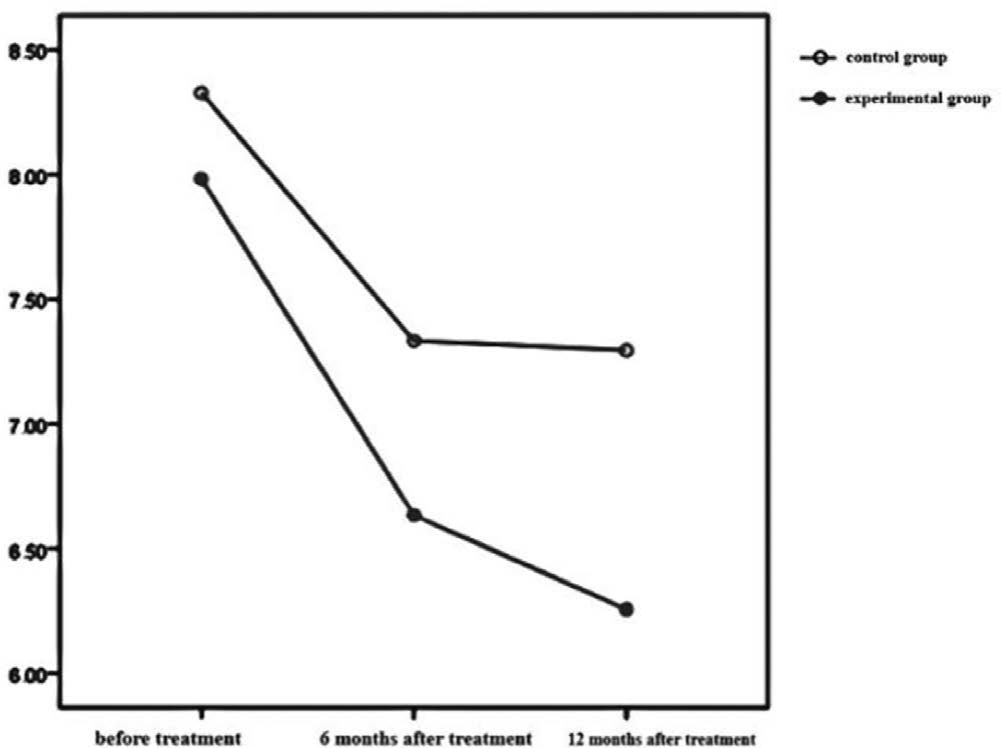
FPG levels at different time points and interaction by group in the 2 groups. FPG = fasting plasma glucose.

The time effects of BMI, HbA1c, and FPG in the 2 groups were statistically significant (*P* < .001), which indicated that BMI, HbA1c, and FPG in the test group and the control group changed with time during the treatment period. The inter group effect of BMI in the 2 groups was statistically significant (*P* < .05), but the inter group effect of HbA1c and FPG in the 2 groups was not statistically significant (*P* > .05). The interaction effect of BMI between the 2 groups was statistically significant (*P* < .001), which showed that the influence of time factors on BMI varied with different treatment methods. See Table 2 and Figures 1–3 for details.

**Table 2:** Comparison of the variable between the 2 groups during treatment.

Variable	Time effect		Intergroup effect		Interaction effect	
	F value	*P* value	F value	*P* value	F value	*P* value
BMI/(kg·m^-2^)	25.449	<.001	4.113	.047	20.854	<.001
HbA1c/(%)	58.087	<.001	3.556	.064	0.225	.724
FPG/(mmol·L^-1^)	22.790	<.001	3.287	.074	1.240	.283

BMI = body mass index, FPG = fasting plasma glucose, HbA1c = Hemoglobin A1c.

### 3.3. Comparison of BMI over time

Further analysis of variables with statistically significant interaction effects over time showed that the BMI level of the test group was lower than that of the control group 6 months after treatment and 12 months after treatment (*P* = .017; *P* < .001). Compared with that before treatment, the BMI level of the test group decreased (*P* < .001), and before treatment >6 months of treatment >12 months of treatment (*P* < α′), There was no statistically significant difference in BMI level in the control group (*P* > α′), see Table 3 for details.

**Table 3:** Comparison of variable with statistically significant interaction effects between the groups during treatment.

Variable	Groups	Number of cases	Before treatment	6 months after treatment	12 months after treatment	F value	*P* value
BMI/(kg·m^-2^)	Experimental group	32	27.77 ± 3.25	26.17 ± 2.60*	25.54 ± 2.22*^,^†	42.011	<.001
	Control group	35	27.74 ± 2.33	27.65 ± 2.33	27.63 ± 2.21	0.129	.813
	t value		0.002	6.026	14.844		
	*P* value		0.966	0.017	<0.001		

*Note*: After correction by Bonferroni method, compare and check the level α′=0.017. BMI = body mass index.

* Indicates that compared with that before treatment *P* < .017.

† Indicates that compared with 6 months after treatment *P* < .017.

### 3.4. Comparison of adverse events

After 12 months of treatment, no hypoglycemia occurred in both groups. There were 3 cases of gastrointestinal reactions in the control group and 4 cases of gastrointestinal reactions in the test group. The symptoms were mild and recovered or improved after symptomatic treatment. The incidence of adverse reactions was 8.57% in the control group and 12.5% in the test group. There was no significant difference in the incidence of adverse reactions between the 2 groups (*P* > .05).

## 4. Discussion

Obesity or overweight, as a chronic disease, can aggravate insulin resistance in diabetic patients, and is closely related to the development of cardiovascular disease and related metabolic diseases.^[1]^ Semaglutide unabated sustainability in treatment of type 2 diabetes and researching cardiovascular events with a weekly incretin in diabetes and other large-scale clinical trials.^[9,10]^ It is confirmed that GLP-1 receptor agonist has dual effects of reducing glucose and weight and reducing the risk of cardiovascular events.^[11]^ In the one-year follow-up observation of this study, the results showed that the long-acting GLP-1 receptor agonist could achieve satisfactory efficacy in overweight or obese patients with type 2 diabetes mellitus. In terms of drug effectiveness, we will further control glycosylated hemoglobin and fasting blood glucose and reduce BMI on the basis of the original hypoglycemic program, which is similar to Li Yanni^[12]^ and Hansen.^[13]^ The results were consistent with previous studies. In terms of drug safety, 4 cases of adverse reactions, mainly gastrointestinal reactions of nausea, abdominal pain, and diarrhea, were observed in this study, which were tolerated by the patients without interruption of the treatment, in line with previous studies.^[14,15]^ The results showed that the adverse gastrointestinal reactions were mild to moderate, and that the mechanism of their occurrence may be due to drug-induced changes in the secretion of peptide hormones in the gastrointestinal tract or indirect effects on the central nervous system.^[16]^

In conclusion, GLP-1 receptor agonists represented by dulaglutide or semaglutide can not only reduce glycosylated hemoglobin and fasting blood glucose levels in overweight or obese patients with type 2 diabetes, but also improve BMI. As a supplement to classical statistical methods, repeated measurement analysis of variance makes full use of the data at each time node in the follow-up process to reflect the change characteristics of observation indicators at different time points relatively objectively, so as to avoid the inability of previous research methods to analyze the internal relationship between the measurement data of the same individual at different time points.^[17,18]^ This study is a single center study, with a small number of cases included. Further research on a randomized double-blind controlled trial with multiple centers and large samples is needed to explore the efficacy of GLP-1 receptor agonist in overweight or obese diabetic patients more comprehensively.

## Author contributions

**Conceptualization:** Wanying Yang.

**Data curation:** Wanying Yang, Lu Wang, Yunhui Zhao.

**Formal analysis:** Wanying Yang.

**Funding acquisition:** Tingyu Ke.

**Investigation:** Wanying Yang.

**Methodology:** Wanying Yang.

**Project administration:** Tingyu Ke.

**Resources:** Xiangming Zhou, Yuanyuan Miao.

**Software:** Xiangming Zhou, Yuanyuan Miao.

**Writing - original draft:** Wanying Yang.

**Writing - review & editing:** Lili Ban.
